# Discrimination and Social Support Experiences for Queer, Rural Adults

**DOI:** 10.1093/geroni/igaf122.1729

**Published:** 2025-12-31

**Authors:** Bryce Van Vleet, Heather Fuller

**Affiliations:** Eastern Mennonite University, Harrisonburg, Virginia, United States; North Dakota State University, Fargo, North Dakota, United States

## Abstract

Minority stress theory (MST) suggests that queer people exhibit worse health outcomes due to increased discrimination. The following study investigates MST and social support in queer, rural adults (age range=18-60) utilizing the TransPop and Generations combined dataset. Rural adults who self-identified as a sexual or gender minority (n = 224) were recruited from across the US. Survey measures included: the Everyday Discrimination Index; mental health (Kessler 6 – Mental Distress); self-rated physical health; and the Multidimensional Scale of Perceived Social Support. ANOVA was used to compare differences in discrimination, health, and social support across three gender categories: cismen, ciswomen, and gender diverse. Only mental health significantly differed by gender (F(2)=3.44, p=.034, 𝜂2=0.02) with gender diverse people (M = 10.63) indicating significantly worse mental health than sexual minority cisgender men (M = 7.82; p=.029). As social support is a touted benefit of rural communities, a mediation was conducted using SPSS PROCESS to test MST. Discrimination was found to partially mediate the relationship between four types of social support (general, significant others, family, friends) and mental health, but not physical health, lending support to MST. Finally, moderation analyses investigating buffering effects of social support on the association between discrimination and mental distress were not significant. These findings suggest that queer identities are mostly equally marginalized in rural areas, but that mental health is particularly poor for gender diverse adults. Results affirm MST, but suggest that queer people in rural areas may not benefit from supportive social networks. Thus, queer, rural adults face unique obstacles across the life span.

